# (In-)formal caregivers’ and general practitioners’ views on hospitalizations of people with dementia - an exploratory qualitative interview study

**DOI:** 10.1186/s12913-017-2484-9

**Published:** 2017-08-04

**Authors:** Nadine Janis Pohontsch, Martin Scherer, Marion Eisele

**Affiliations:** Department of General Practice/Primary Medical Care, University Medical Center Hamburg-Eppendorf, Martinistr. 52, 20246 Hamburg, Germany

**Keywords:** Dementia, Hospitalization, Prevention, Interview, Qualitative research

## Abstract

**Background:**

Dementia is an irreversible chronic disease with wide-ranging effects on patients’, caregivers’ and families’ lives. Hospitalizations are significant events for people with dementia. They tend to have poorer outcomes compared to those without dementia. Most of the previous studies focused on diagnoses leading to hospitalizations using claims data. Further factors (e.g. context factors) for hospitalizations are not reproduced in this data. Therefore, we investigated the factors leading to hospitalization with an explorative, qualitative study design.

**Methods:**

We interviewed informal caregivers (*N* = 12), general practitioners (GPs, *N* = 12) and formal caregivers (*N* = 5) of 12 persons with dementia using a semi-structured interview guideline. The persons with dementia were sampled using criteria regarding their living situation (home care vs. nursing home care) and gender. The transcripts were analyzed using the method of structuring content analysis.

**Results:**

Almost none of the hospitalizations, discussed with the (in-)formal caregivers and GPs, seemed to have been preventable or seemed unjustifiable from the interviewees’ points of view. We identified several dementia-specific factors promoting hospitalizations (e.g. the neglect of constricted mobility, the declining ability to communicate about symptoms/accidents and the shift of responsibility from person with dementia to informal or formal caregivers) and context-specific factors promoting hospitalizations (e.g. qualification of nursing home personal, the non-availability of the GP and hospitalizations for examinations/treatments also available in ambulatory settings). Hospitalizations were always the result of the interrelation of two factors: illnesses/accidents and context factors. The impact of both seems to be stronger in presence of dementia.

**Conclusions:**

Points for action in terms of reducing hospitalization rates were: better qualified nurses, a 24-h-GP-emergency service and better compensation for ambulatory monitoring/treatments and house calls. Many hospitalizations of people with dementia cannot be prevented. Therefore, hospital staffs need to be better prepared to handle patients with dementia in order to reduce the negative effects of hospitalizations.

**Electronic supplementary material:**

The online version of this article (doi:10.1186/s12913-017-2484-9) contains supplementary material, which is available to authorized users.

## Background

Dementia is an irreversible chronic disease with wide-ranging effects on afflicted persons’, caregivers’ and families’ lives. Extrapolations expect 115 million of new patients to be affected worldwide by 2050 [1]. Hospitalizations are significant events for people with dementia. They tend to have poorer outcomes compared to those without dementia. Mortality rates are twice as high for people with dementia and the risk of delirium is increased [2]. Transfer, unfamiliar surroundings and unknown medical personnel can be confusing and anxiety provoking for patients with advanced dementia [3] and lead to an aggravation of cognitive deficiencies [4]. People with dementia are more likely to be admitted to the hospital than comparable people without dementia [5–7]. This is especially the case for ambulatory care-sensitive conditions (AC-SC) [6]. Dementia was not the reason for the increased admission rates [5], therefore some of these hospitalizations might be considered unnecessary or preventable. As hospitalizations are stressful events for people with dementia, they should be prevented if the appropriate medical treatment is also available in ambulatory care [7].

Most of the previous research focused on diagnoses leading to hospitalizations using claims data (e.g. [7, 8]), but claims data is limited because it represents the “billing reality” but not the “treatment or care reality”. Other studies only tested a limited number of anticipated influence factors and, therefore, only shed light on some parts of the bigger picture. The situation of caring for a person with dementia, be it informal care at home or formal care in a nursing home, is very complex and involves different stakeholders (e.g. informal caregivers including family members and other associates, e.g. friends, the general practitioner (GP) and formal caregivers, e.g. nursing staff).

We assume that further relevant factors leading to hospitalizations are not reproduced in recent studies and can only be investigated in an exploratory, qualitative study design. In this study, we investigated the question as to whether or not some of the hospitalizations were considered preventable from the interviewees’ points of view. If so, we aim to identify possible starting points for preventing unnecessary hospitalizations. To take the complex care situations into account, we considered the perspectives of informal caregivers, GPs and formal caregivers of people with dementia.

This qualitative interview study aimed to answer the following questions: How do informal caregivers and professionals (GPs and formal caregivers) describe the circumstances and reasons leading to a hospitalization of a person with dementia? How do they feel about the necessity or preventability of these hospitalizations? Which factors contribute to the hospitalization of people with dementia? What can be done to reduce the hospitalization of people with dementia?

## Methods

Given our objective to investigate the circumstances of hospitalizations of persons with dementia and views on preventability of these hospitalizations, we chose to conduct an exploratory study using qualitative research methods. We performed semi-structured interviews with a dyad or, if available, triad of informal caregivers (family members and other associates, e.g. friends), GPs and formal caregivers (nursing staff) of 12 persons with dementia. The study was funded by the German Research Foundation (reference number: EI 955/1–1). Ethical approval was obtained from the ethics committee of the Medical Chamber Hamburg (May 13th, 2013; PV 4339).

### Participants and recruitment

We chose to interview informal caregivers, GPs and formal caregivers of 12 persons with dementia in order to obtain a comprehensive view on the hospitalizations of people with dementia to enhance the understanding of interactions and to gain insight in different individual needs [9]. The persons with dementia were sampled [10] according to the empirically derived criteria: living situation and gender, both of which have been shown to be influential on hospitalization rates [8, 11].

Inclusion criteria for the persons with dementia were: a medical diagnosis of dementia and having been admitted to stationary treatment in a hospital during the last 12 months. They were identified from an existing cohort of people with dementia (AgeCoDe cohort established in 2003/4, e.g. [12]). Relatives of eligible persons with dementia (both included in the AgeCoDe-Study), who gave their consent to be contacted for other studies, were asked to participate in our study. If the informal caregiver was interested in participating in the study, written informed consent for interviewing the informal caregiver, the GP and the formal caregiver (including the release from his/her obligation of confidentiality) was obtained, either from the person with dementia (if able to consent) or his/her legal custodian. Then the GP and formal caregiver(s) were invited to take part in the study. All interviewees gave their written informed consent to be interviewed, for the interview to be digitally recorded and for it to be used for the study.

Since we could not include enough persons with dementia (*N* = 5) from the AgeCoDe cohort, we contacted all GPs participating in the AgeCoDe study requesting them to take part in the study by identifying patients fulfilling the inclusion criteria and contacting the informal caregiver to invite them to participate in the study. If the informal caregiver was interested in participating in the study, they were included in the study as described above.

The characteristics of the persons with dementia, interviews and interviewees are displayed in Table 1. We conducted 12 interviews with informal caregivers (family members), 12 interviews with GPs and 5 interviews with formal caregivers (paid staff). The majority of the interviews with informal caregivers (*N* = 10) were conducted in the caregivers’ homes, one was conducted via telephone (because the relative was living in another federal state) and one was conducted at the Department of Primary Medical Care. The interviews with the GPs were conducted at the GPs’ offices (*N* = 7), by phone (*N* = 4) (which is regarded acceptable for expert interviews [9]), or at the GP’s home (*N* = 1). Interviews with formal caregivers were all conducted at the interviewees’ work places. The interviews lasted between 30 min and 2 h. All interview quotes in the results section were translated from German by a native speaker and checked for accuracy by NP.

**Table 1:** Characteristics of persons with dementia and interviewees

Person with dementia’s situation of living (N; female/male)	• Private apartment (6; 3/3) • Nursing home (6; 3/3)
Informal caregivers’ relation to person with dementia (N; female/male)	• Former spouse (1; 1/0)/spouse (2; 2/0)/partner (1; 0/1) • Child (5; (2/3)/child-in-law (2; 2/0)
Interview mode for general practitioners (N; female/male)	• Face-to-face (8; 4/4) • By phone (4; 1/3)
Interview mode for formal caregivers (N; female/male)	• Face-to-face (5; 4/1)
Number of hospitalizations discussed, N (Median)	• 1–7 (3)

### Interview conduction

All interviews were conducted using a semi-structured interview guide [13]. For guidelines see Additional file 1. As our study was designed to be exploratory, this approach allows the interviewer to ask individualized questions deviating from the prescripted questions, if needed, to explore new or unexpected topics brought up by the interviewee. All interviews were conducted by NP (psychologist, postdoctorate research fellow) between September 2013 and June 2014. We recorded the interviews digitally and transcribed them verbatim. Any personal information that might allow or facilitate to identify a patient (e.g. name of a hospital) was changed during transcription process. The accuracy of the transcripts was checked by NP.

The interview guide differed for informal caregivers, GPs and formal caregivers. All in all, the following topics were covered when applicable: the constitution/health of the person with dementia; the daily routines/everyday life/usual duties in caring for the person with dementia; the (access to) ambulatory care; the nursing service support’s contact/communication with the GP; medical care in the nursing home; contact/communication of informal caregivers and formal caregivers/nursing home; contact/communication (of informal and formal caregivers) with the GP; the GP’s relationship with the patient; the GPs’ contact/communication with the informal/formal caregivers; the (necessity of) hospitalization(s) from the interviewee’s point of view; desiderata for care and ideas for preventing hospitalizations. There was no data obtained from medical records by the interviewer.

### Analysis

The transcripts were analyzed using the method of structuring content analysis. The goal of the structuring content analysis is to structure (and summarize) the content of the interviewees accounts during the semi-structured interview [14, 15]. This procedure extracts and preserves the essential content of the data, while significantly reducing the amount of data.

All transcripts were read several times before coding. Deductive categories were derived from the research questions and the interview guidelines. During the review of the interview material deductive categories were supplemented by inductively formed categories. Due to the exploratory nature of the study, the focus was placed mainly on the inductive category formation. Transcripts were broken down into fragments of analysis, each containing one idea. These fragments can adopt different sizes ranging from parts of a sentence to a whole paragraph. If a fragment relevant to the research question was identified, a category name was derived from this fragment and a description of the category was drafted and supplemented by an exemplary quote [14].

We used the same category system to code the interviews from all sources. NP coded all the material in close consultation with ME (psychologist, post-doctorate research fellow), the relevance of the categories and codes were discussed multiple times throughout the research process. Anonymized data and its analysis were discussed in two different research workshops to assure an intersubjective comprehensibility of the analysis. After coding all the material, a second round of coding was performed to ensure that no relevant information was missed. Data was managed using MAXQDA 10 (Verbi GmbH).

## Results

The results section is divided into four main sections. The first section ‘circumstances and reasons for the hospitalization of persons with dementia’ shall inform the reader about the context and nature of the hospitalizations discussed during the interviews to better understand the following depiction of the main categories answering our research questions. This section is followed by the description of the main categories ‘preventability of hospitalizations’, ‘factors contributing to hospitalizations’ with its subcategories ‘dementia-specific factors’ and ‘context-specific factors’, and ‘ideas for reducing hospitalizations’. Table 2 gives an overview of the main and subcategories.

**Table 2:** Overview of main and subcategories

1) Context and nature of hospitalizations in our study	• Planned treatments/operations	
	• Unplanned treatment	• Aggravation of the general condition • Exsiccosis • Ealls in the nursing homes/at home • Other conditions
2) Preventability of hospitalizations	• Most hospitalizations not preventable/unjustifiable from interviewees point of view	
	• GPs strive to prevent hospitalizations wherever possible	
	• Informal caregivers do not see themselves in a position to decide about the necessity of a hospitalization	
3) Factors contributing to hospitalizations		
• Dementia-specific factors	• Agitation/restlessness	
	• Tendency to stray/tendency to run away	
	• Neglect of restricted mobility	
	• Declining ability to communicate about symptoms (and accidents)	
	• Shift of responsibility from person with dementia to informal or formal caregivers	
• Context-specific factors	• Nursing-home-specific factors	• Safeguard against legal consequences
		• Qualification of nursing home staff/resident-nurse-ratio
	• Non-availability of the GP	
	• Hospitalizations for examinations/treatments also available in ambulatory settings	
	• Communication (problems/lack of communication)	
• Interrelation between dementia- and context-specific factors		
4) Ideas for reducing hospitalizations	• Qualification of formal caregivers in nursing homes	
	• Twenty-four-hour-GP-emergency service	
	• Adequate compensation of regular home visits and supporting visits from ambulatory care services	

### Circumstances and reasons for the hospitalization of persons with dementia

Conditions leading to hospitalization were manifold and can be categorized as: planned treatments/operations, unplanned treatment due to the aggravation of the general condition or due to exsiccosis, falls in the nursing homes, falls at home and other conditions. Table S1 shows examples [see Additional file 2].

Some of the hospitalizations were due to planned treatments like the insertion of a cardiac pacemaker or stents, the treatment of an eczema, eye surgery, post-therapeutical check-ups on arterial occlusion, or a operation on a patient taking coagulation inhibitors. Other hospitalizations were described as due to exsiccosis (either because of vomiting, diarrhea or insufficient fluid intake) or a general aggravation of a condition of the person with dementia.

Many incidences of falls in nursing homes were reported. Although not every fall automatically results in a person’s hospitalization, all hospitalizations due to falls in nursing homes were instigated by formal caregivers calling the ambulance either because of detectable injuries or to make sure that no injuries were missed. Reasons for the falls were manifold, for example: slipping on a wet spot, neglecting restricted mobility (further explanation below) or cardiac arrhythmia. Sometimes the reasons were unknown: The person was found on the ground, but, due to dementia, was not able to explain what happened. Falls of persons with dementia still living at home often happened in- or outside of the home while they were alone or unattended. Especially in persons with dementia living alone in their own apartment, the circumstances surrounding the falls could not always be uncovered. In most situations the (in-)formal caregivers or other persons called the ambulance, in one case the person concerned called the ambulance by himself, after contacting the informal caregiver. In another case, the person concerned was admitted to the hospital by the GP one day after the accident occurred. Examples for circumstances surrounding the falls were: hypoglycaemia, having left outpatient care service centers unattended (with restricted mobility), induced by exsiccosis or medication, neglect of restricted mobility or bicycle accident.

Besides the above mentioned circumstances, various other reasons for hospitalizations were reported: Bloody vomiting/oesophageal ulcers, bloody stool, gastrointestinal infection (with fever), intestinal infections, suspected stroke, hypoglycaemia, cardiac arrhythmia, abdominal complaints/pneumonia and biliary tract inflammation.

### Preventability of hospitalizations

Almost none of the hospitalizations, discussed with the informal caregivers and GPs, seemed to be preventable or unjustifiable from their point of view. This is not only true for those incidents in which the interviewed person was directly responsible for the hospitalization (e.g. in the sense of being the admitting practitioner or the caregiver calling an ambulance), but also for almost all hospitalizations induced by others. While GPs reported trying to prevent hospitalizations, informal caregivers acknowledged potentially harmful effects of hospitalization but as medical laypersons did not see themselves in a position to decide about the necessity of a hospitalization. GPs almost never mentioned that an informal caregiver made a ‘wrong decision’ by calling an ambulance.

Interviewer: And would you, in your opinion, say that it was necessary that she was admitted to the hospital after this incident? General practitioner: Yes, with unconsciousness and after collapsing, one should take a look. There is always the possibility of it being a heart attack or a stroke which you can only clear up in a hospital.

Informal caregiver: Definitely, yes, definitely. There are situations where a person with dementia falls or becomes ill with something that the nursing home or the private home environment cannot evaluate or are not equipped to deal with. I am completely convinced that I’d be the person to 100% support sending these people to the hospital quickly even if a doctor just quickly takes a look to make sure everything’s ok […].

### Factors contributing to hospitalizations

#### Dementia-specific factors

The following dementia-specific factors seem to be influential concerning hospitalizations. While the first three: agitation/restlessness, the tendency to stray/run away and neglecting restricted mobility primarily raise the risk for hospitalizations due to fall-related injuries, the limited ability to describe or communicate about symptoms/accidents is a potential risk factor for hospitalizations due to all kinds of illnesses/conditions. Shift of responsibility from person with dementia to informal or formal caregivers influences the decision process regarding hospitalizations.

#### Agitation/restlessness

Relatives, GPs and formal caregivers often mentioned the heightened urge for activity of people with dementia living both in private and in nursing homes. This heightened, often undirected urge for activity seems to hold a great risk for falls and fall-associated injuries. On the one hand, this may be due to sedating medications given to the patient (thereby increasing the risk of falls), or, on the other hand, may be due to persons’ failure to use required walkers or canes (see also ‘neglect of restricted mobility’).

Formal caregiver: […], that she fell and had that facture, she automatically gets classified as at high risk for falls […] So a walker was ordered for her, but she doesn’t use it. […] animate her not to walk too quickly, because sometimes she downright dashes down the hallway.

#### Tendency to stray/tendency to run away

Many persons with dementia show, at least in early stages of the illness, a tendency to stray or run away from their private or nursing homes. Often they do not find their way back to their (nursing) home and have to be searched for or brought back by the police. Combined with an often reduced mobility or disorientation, this tendency to leave their (nursing) homes poses another risk for hospitalizations due to fall-related injuries or other medical conditions.

Formal caregiver: […] And the other [day care center] is far more open and he ran away without coming back at all more often. This also caused the fall, which led to him having to be hospitalised. […]

#### Neglect of restricted mobility

Sometimes persons with dementia seem to forget their physical limitations like decreased mobility. Formal caregivers report that this, too, results in falls. For example, falls occur when they try to get out of bed, although they are not able to do so without help or aids.

Formal caregiver: […] That one time she fall pretty badly […]. She had degenerated significantly, especially physically. She couldn’t stand up alone anymore, as I said before, she forgot that she couldn’t stand up on her own. […]

#### Declining ability to communicate about symptoms (and accidents)

Many interviewees reported that people with dementia have a reduced ability to communicate with other people in a meaningful and constructive way. This naturally also applies to communication about their state of health or symptoms experienced with informal and formal caregivers, GPs, other physicians or paramedics. Some of the patients did not remember how they got injured, whether they had fallen or which illnesses they have. If communication deficits are further advanced, people with dementia may no longer be able to communicate with the physician at all. Anamnestic self-report is, therefore, hindered or useless, for example, because pain is not reported. Physicians often have to rely on reports from (in-)formal caregivers. This may result in hospitalizations to examine whether the reasons for the symptoms are harmless or serious.

Formal caregiver: […] Especially in people with dementia, the problem exists that they cannot express pain in detail so that an X-ray is necessary to see where the pain comes from and where something might be broken.

General practitioner: […] if there had been afflictions, it is likely that she would not have been able to interpret these. […] If one asked her ‚Do you have heartburn?,‘ I think she would probably say ‚no‘ but wouldn’t even know what that is.

#### Shift of responsibility from person with dementia to informal or formal caregivers

Most interviewees describe a shift in responsibility for appropriate medical care (the organization and keeping of appointments, the communication with the GP and other physicians) and medication intake from the persons with dementia to (in-)formal caregivers, even in the initial stages of dementia. It was striking that none of the interviewees mentioned having discussed the need of calling an ambulance or an emergency physician with the persons with dementia. Decisions seemed to be made without consulting the patient even in the initial stages of dementia. As the dementia changes for the worse, the ability to coordinate doctor’s appointments and medication intake decreases and other people take over these responsibilities. The person with dementia seems to lose his/her self-management abilities and ceases to be a responsible patient. Informal caregivers of persons with dementia still living in private homes take over increasing responsibility for coordinating the patient’s medical care (e.g. arranging appointments, controlling medication intake and deciding about which kind of help to get in case of emergencies or health deteriorations).

General practitioner: […] He [the informal caregiver] calls sometimes, because we talk on the phone intermittently. I ask him to call me back when we give diuretic medications, then he tells me about the process, whether she, how the weight developed. He comes by to pick up prescriptions […], when they come together, he is the one who leads the conversation. […]

Informal caregiver: It’s just that she didn’t do it herself anymore. So I apportioned her pills and had them for her, there are containers with days of the week, not days of the weeks but morning, noon, and evening. […] And so I did that for her, well, and later, when she couldn’t do it on her own anymore, the ambulatory care nurses took care of it. I would apportion the pills and ask the ambulatory care nurses to ‘please keep an eye on it as well’. Or I checked in the mornings myself.

Informal caregivers are mostly medical laypersons and might, therefore, be overprotective or unsure whether the patient’s symptoms need emergency treatment or not. For patients living in nursing homes, the nursing home employees take over these responsibilities.

General practitioner: […] or the attention from family members that care so much about the affected person, that they immediately call the air ambulance. […]

### Context-specific factors

#### Nursing home-specific factors

The following two codes, ‘safeguard against legal consequences’ and ‘qualification of nursing home personal/resident-nurse-ratio’, are nursing-home-specific and only apply for nursing home residents. They seem to be interrelated, as interviewees often indicated that the nursing home has to safeguard itself against legal consequences of not reacting adequately to injuries or health problems of their residents. The lower the qualification of the nursing home staff and the higher the workload, the faster they seem to call ambulances and induce transfers to the hospital just to be on the safe side.

#### Safeguard against legal consequences

Interviewees from each group (informal caregivers, GPs and formal caregivers) mentioned the perceived need of the nursing home staff to safeguard themselves against legal consequences of not reacting adequately to falls, injuries or health deterioration of their clients. After-hours emergency services or ambulances are called, because one ‘cannot determine whether he/she really needs to be brought to the hospital or not’. This may result in some unnecessary hospitalizations.

Interviewer: And would you, in your professional opinion, say that they always make the right call or do they call the ambulance too often or…? General practitioner: Well, they naturally have to call the ambulance when in doubt out of legal reasons.

General practitioner: […] In one ward, every time [a patient] bumped into something or fell down and stood back up without any complaints, he was sent to the hospital for legal reasons, because of the home supervisory authorities. So in one year about 35 times. […]

#### Qualification of nursing home staff/resident-nurse-ratio

GPs attributed some, possibly unnecessary, hospitalization to the lacking qualification of some of the nursing home personnel. The less educated the personnel is, the higher the likelihood that an after-hours emergency service or ambulance is called, even in mild or harmless cases of health deterioration. Informal caregivers and GPs also often mentioned that there doesn’t seem to be enough personnel in the nursing homes or a high staff fluctuation, resulting in everyone having very little time, not really knowing the residents and higher educated personnel not being present.

General practitioner: You simply need someone, who will sign for it … who will take responsibility and stick their neck out in case something goes wrong. And the less educated and the more overwhelmed they feel, they’d naturally rather call the ambulance one too many times than once too late.

General practitioner: […] it very much depends on the individual, on the situation, since they [the nurses] carry a lot of responsibility and are not necessarily qualified to do certain things or decide certain things, while being under a lot of pressure not to make any mistakes. Because of this pressure not to do anything wrong a patient is sent to the hospital more often […].

#### Non-availability of the GP

After-hours emergency service or an ambulance is called by formal caregivers in times when the GP is not available (for a house call). On some occasions a house call by the GP could have prevented the hospitalization because they know their patients and their medical conditions and can better decide if the situation is serious enough to require hospitalization. Physicians from the after-hours emergency service do not know the patients and their medical history, are under time pressure, and may have no specialization on family medicine, thus, respectively little experience with geriatric patients and patients with dementia. Because of a lack of information, they might rather decide for hospitalization than for a “wait and see”- strategy that the GP might have chosen.

General practitioner: Yes, because we know them. The after-hours emergency service doesn’t know the patients. It’s hard to ask a patient with dementia about their medical history or about known illnesses, thus, they have to depend on the documentation kept in the nursing home and the diagnoses in the nursing home’s computer. But they are, for example, missing all the hospital reports from earlier stays and different hospitals. Generally they only see the most recent hospital report so that we simply know the patients better and also know if something similar had already occurred before, what the cause was, how it was treated, how it went away… All things that the emergency service cannot know. Naturally, the emergency service physicians have to depend on their senses then and, if they cannot figure out what is wrong, the only option left is a hospitalization to identify the issue.

Interviewer: And would you say that it was a necessary hospitalization? General practitioner: It could have been avoided, if they had somehow reached me at 7 p.m. or 8 p.m., then I would have come by after my practice hours and then she would have received MCP and could have stayed at home. […]

#### Hospitalizations for examinations/treatments also available in ambulatory settings

In some cases hospitalizations seem to be necessary, albeit examinations or treatments could also be done in an ambulatory setting. This might be due to the patient’s comorbidities, which do not allow an ambulatory treatment (because further monitoring is needed) or because multiple examinations are necessary, which cannot be accomplished by the patient in an ambulatory setting due to his mental and physical state. Another reason mentioned in the interviews is that, in the case of dehydration, intravenous fluid replacement is only possible in the hospital and is preferred to the subcutaneous fluid replacement possible in the nursing home. In case of examinations (like x-rays after a fall), which can possibly lead directly to further treatment needs (for example an operation), it is easier and cheaper to conduct them directly in the hospital. In other cases, patients with dementia are sometimes not so easy to handle (causing problems with their safe transportation or waiting times in ambulatory settings), so that transporting the patient in an ambulance and examining them in a hospital seem to be the easiest way to handle their health problems.

General practitioner: So the [hospitalisations] that I experienced were okay, because he needed an infusion and then one needs to make sure that the patient gets back on his feet quickly. Um, and in this case, it was surely best to use an intravenous fluid replacement method because otherwise everything would have taken much longer. I feel… that subcutaneous fluid substitution is not as good, some facilities do this alternatively. […] And especially when it’s an emergency situation, then something needs to be done quickly and intravenous substitutions are simply not possible in nursing homes, because there is no one there qualified to monitor them.

Formal caregiver: […] for example when I see someone with a foot like that in the morning, then I have to do something. Then the problems start. I could say, ok, I’ll take them to the GP, he’ll send her in for an X-ray, they check out what’s wrong or sometimes it goes right to the surgeon’s, […]. In hospitals everything is available, if the patients are sufficiently chaperoned there, it is much easier to do it that way. But, for example, with a (person B) you can’t –even if the son were on board- say, ok- I’ll take her in my car and drive her to the doctor’s office, I... […] You need at least two people. Because, at least while (person B) was still mobile, if they stopped at a crossing, at a red stoplight somewhere and she felt like it, she would just get out of the car. Or you have your car somewhere, she runs off, and you can call after her, but no. And she’s fast, very fast, well not anymore unfortunately but, well. So, I guess, that then (...) exams, where she was in the (hospital), could have been avoided with enough chaperoning- if two people were available. But exams in the clinic are much easier. That needs to be said.

#### Communication

Besides the abovementioned communication problems based on the persons’ with dementia declining ability to communicate adequately, many other communication problems were mentioned by the interviewees. These communication problems might be more relevant because the person being cared for has only limited or no abilities left to coordinate and manage their health care. Communication problems or non-communication occur at all interfaces, for example between the GP and the nursing home or ambulatory care services and between the GPs and the informal caregivers.

The amount and quality of the communication between GPs and nursing home employees seem to depend on whether the physician looks after only one patient, after a small number of patients or after a larger number of patients in the same nursing home. For both, the nursing home employees and the GPs, it is easier to exchange information about patients if the GP visits several patients in one nursing home on a regular basis (at times jointly agreed upon). In this case visits can be prepared for by the nurses and a qualified nurse can act as contact person and information provider during the visits.

General practtioner: Yes, well I find the responsible nurse or nursing assistant and we sit down together. Then I don’t have to visit the patients that I’ve known for a long time, where the nurses say: ‘Nah, everything is stable, the same as always.’ It’s a bit of a judgement call. […] Then we exchange information and it’s basically like a visitation, well it is a visitation.

GPs and ambulatory care services seem to generally have little to no contact; therefore, an exchange of information about the state of health of the person with dementia does not take place. Only one exception was reported by a GP, describing monthly to quarterly meetings, with representatives of an ambulatory care service, to discuss common patients.

General practitioner: Exactly, we have our main ambulatory care service, […] with whom we meet every four weeks or, as of late, every quarter. The main ambulatory care service caregiver and their boss come and we simply go through and discuss all our common patients together with my assisting physician, who does more of the house calls and partially knows the patients better than I do.

Some of the GPs mentioned that close contact with the informal caregivers (caring for persons with dementia at home) would give the benefit of having more accurate information about the state of health and the need of support of the person with dementia. Usually such close contact cannot be achieved during the hectic, daily office routine. It is also perceived to be the caregiver’s responsibility to initiate contact or information exchange. One informal caregiver reported that she does not really know which medications the person with dementia takes and whether there may be side effects.

General practitioner: One should have a regular appointment in during practice hours, every quarter, where one can, can compare what he says and what she says. In some cases it’s two separate worlds, and in some cases it might have been interesting. […] Anyway, then [the initiative] would have had to come from the family members. And that, yes that is difficult. That, because that for me, was incredibly difficult for me to see during these very short consultations.

Interviewer: But you didn’t, let me say, exactly know what all those pills are for? Informal caregiver: Nah, nah, yea I always had to read through [the information] and they were often for the heart. […]

#### Interrelation of dementia-specific factors, context-specific factors and illnesses/accidents

**Fig. 1:**
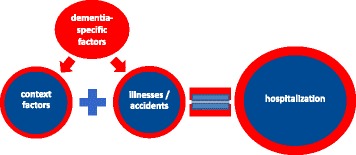
Relationship between context factors, illnesses/accidents and hospitalizations enhanced by dementia-specific factors

Figure 1 depicts hospitalizations as the result of the addition of two factors (shown in blue): the occurrence of illnesses/accidents (as described in the section ‘circumstances and reasons for the hospitalization of persons with dementia’) and the context factors under which the illnesses/accidents occur (as described in the section ‘context-specific factors’). Dementia-specific factors have an impact on both, illnesses and accidents and context-specific factors. Accidents seem to happen more often under the presence of dementia-specific factors like agitation and restlessness, the tendency to stray/run away and the neglect of restricted mobility. The declining ability to communicate about symptoms/accidents and the shift of responsibility from the person with dementia to (in-)formal caregivers increases the impact of context factors like the unavailability of an GP, nursing home-specific factors or all-present communication problems. All in all, the presence of the dementia-specific factors increases the impact of both, context factors and illnesses/accidents, on the probability to be hospitalized. The impact of the presence of dementia-specific factors is shown in red in Fig. 1.

### Ideas for reducing hospitalizations

Due to the fact that most of the hospitalizations were not rated as preventable or unnecessary by the interviewees, only few ideas as to how to possibly reduce hospitalizations came up during the interviews. GPs and formal caregivers mentioned some ideas, while informal caregivers mentioned no ideas as to how to reduce hospitalizations.

Nevertheless there were some topics mentioned by the GPs regarding the prevention of hospitalizations. A recurring topic was the already mentioned qualification of the caregivers in nursing homes, higher education being equated with a better performance in judging the residents’ state of health and need for further medical care. Some GPs mentioned the idea of a 24-h-GP-emergency service. The continuous relationship between a GP and a patient, characterized by the GP’s good knowledge of the patient and his medical and personal history, facilitates correct judgment about the actual state of health and the need of hospitalizations. This could be implemented as an emergency service delivered by several GPs for specified nursing homes, where the patients are known to all the doctors.

General practitioner: The (name)-ambulatory care network is modelled after procedures in (city) and is being planned for some nursing homes here as well. That an around-the-, around-clock-, so, 24-hour GP care is to be available, that you [the GP] are constantly available via cellphone and are supposed to come right away. That was…, probably against the background of ‘reduction of hospital stays’, I think so, yes. […]

The formal caregivers also mentioned the prolonged availability of a GP, who knows the patient well, as a potential means to reduce hospitalizations.

Formal caregiver: So in the evenings when the GP is not available […], or on Wednesdays when the practices are closed and no home-visits are planned. Then we have to, our only option is to call there [emergency medical service], and that’s what we do.

Interviewer: Is it equally as good as if the GP comes?

Formal caregiver: No, it always depends on the type of physician. There are, in the emergency medical service […], if someone has the flu or pneumonia and a gynecologist comes to our nursing home. […] or it can be a pediatrician. […] It used to be different, but now it’s more often the case that when the physicians themselves are also unsure, they send the patients to the hospital as well. […]

Some GPs stated that hospitalizations might also be prevented if regular home visits and supporting visits from ambulatory care services were adequately compensated (e.g. monitoring acute respiratory diseases, caring for chronic wounds or therapy against hyperhydration). This would mean that the patient could be treated at home and does not have to be transferred to the hospital.

General practitioner: It is certainly true that we send a lot to the hospital because we cannot deal with the problem or because we cannot visit the patient every day because we aren’t paid for it. We simply can’t. […] Dementia in combination with chronic wounds for example, if we could make it there regularly to look at the wound […]. We just had a case, it (the wound) became larger despite the nurses’ care and recommendations following photo documentation. Has to be sent to the hospital for two weeks to receive intensive care there. Perhaps, but we cannot be sure, if we had been there more often, it would have been better. It just isn’t possible to visit more often than every two or three weeks.

## Discussion

### Main findings

We identified several dementia-specific and context-specific factors promoting hospitalizations. Dementia-specific factors were: agitation/restlessness, the tendency to stray/run away, the neglect of restricted mobility, the declining ability to communicate about symptoms (and accidents) and the shift of responsibility from the patient to informal or formal caregivers. Context factors included: nursing home-specific factors, which are only relevant for people living in nursing homes (safeguard against legal consequences, qualification of nursing home personnel/resident-nurse-ratio); and general factors like the non-availability of the GP; hospitalizations for examinations/treatments also available in ambulatory settings; and communication. Almost none of the hospitalizations discussed with the informal caregivers and GPs seemed to have been preventable or were unjustifiable from the interviewees’ point of view.

### Strengths and weaknesses

This is the first qualitative study analyzing possible reasons for hospitalizations of people with dementia. Three different viewpoints on situations leading to hospitalizations of persons with dementia were explored, this reflects the change from the dyadic physician-patient interaction to the triadic physician-(in-)formal caregiver-patient interaction throughout the course of dementia [16, 17]. This inclusion of different viewpoints distinguishes our study from other studies (e.g. [18]) concerning potentially avoidable hospitalizations.

Limitations of this study include the fact that due to the study design only persons with dementia having informal caregivers were included. Therefore, the results cannot be transferred to the population of people with dementia without informal caregivers. Problems might be different here. In addition, it cannot be ruled out whether only informal caregivers with a good relationship to the GP were willing to take part in the study or whether physicians selected persons with dementia with unquestionable hospitalizations.

The design of this study does not enable us to resolve whether all hospitalizations, discussed in the interviews, were objectively unavoidable or whether at least some cases could also have been treated in an ambulant setting. We have no reason to believe that the interviewees have been dishonest about their perception of the avoidability of the discussed hospitalizations. We, therefore, assume that the people concerned did not see any other way to resolve the discussed situations but to take measures eventually leading to the hospitalization of the people with dementia.

Another limitation might be that the GPs often had to rely on hospital reports to provide information during the interview, relatives did not know exact details about hospitalizations if the person with dementia lived in nursing homes, and formal caregivers also often relied on documentation made by colleagues. Nevertheless, the interviews did reveal plenty of information about the hospitalizations of the persons with dementia and about hospitalizations in general.

### Findings relative to other studies

#### Preventability of hospitalizations

In a qualitative study conducted by Freund and colleagues [18], about 59% of all hospitalizations for ambulatory-care sensitive conditions (AC-SCs), in a population at high risk of re-hospitalization, were deemed to be unavoidable by the treating GPs. Many studies report higher numbers of hospitalizations for people with dementia especially for AC-SCs [6]. Our and Freund et al.’s findings, taken together, seem to challenge the assumption that many AC-SC-hospitalizations might really be avoidable (for people with dementia).

#### Factors contributing to hospitalizations

Our study has shown that some dementia-specific factors, like the restricted ability to communicate about symptoms and events leading to injuries, might contribute to the higher hospital admission rate of people with dementia in comparison to people without dementia. Other studies also show that people with dementia have a higher risk of having undiagnosed diseases [19] and of underreporting adverse drug effects [20]. Toot and colleagues state that people with dementia might have a reduced or delayed help-seeking behavior due to a reduced recognition of symptoms or the impaired ability to communicate symptoms. Furthermore, caregivers might misinterpret signals of illnesses in people with dementia leading to strong symptom aggravation before treatment initiation [21]. Other studies support these findings and the authors conclude that practitioners (and caregivers) should have a heightened awareness for possibly underreported symptoms of non-cognitive disorders [22, 23] and adverse drug effects in these patients [20].

Thorpe et al. state that the ability to independently manage medication is among the first functional losses reported for people with dementia, increasing their reliance upon caregivers for assistance with medication [24]. As the interviewed informal caregivers were often responsible for remembering to administer medications and monitoring for adverse events and side effects, but reported insufficient communication about medication, medication education for caregivers (concerning dosage, indication, administration with other medications, recognition of side effects and therapeutic outcomes) would be helpful. This has also been suggested by Campbell and colleagues [25]. Regular drug reviews with the participation of the (in-)formal caregivers and the persons with dementia might be even more necessary for people with dementia than for the general population. This is also demanded by existing guidelines [26] and viewed as an indicator of the quality of care [27]. The interviewed (in-)formal caregiver took over responsibility for the health and medical care of people with dementia. Other studies also state (in-)formal caregivers serve as surrogates for medical decision making among patients with dementia [24], act as care coordinators, and as information sources and front-line communicators [17]. In triads composed of practitioners, people with dementia and informal caregivers interaction shifts over time. Communication with the caregiver increases while communication with the patient decreases [28]. This was also true in our study. The person with dementia is often marginalized in communication and no longer takes part in his/her own care decisions [28], e.g. in our study, the perceived necessity of a hospitalization was never reported to be discussed with the person with dementia. Nevertheless many informal caregivers do not know enough about available help/services [29] and do not get sufficient symptom management advice [30]. This increased need for information is a long known fact [16, 31, 32]. Creating possibilities for the reimbursement of conversations between GPs, informal caregivers and people with dementia (about medications, side-effect-monitoring and health care strategies) could potentially diminish this problem. In addition, a regular exchange between ambulatory care providers and GPs, as well as, between nursing home caregivers and GPs should be fostered, as suggested by our interviewees and van den Bussche and colleagues [33]. This need for the interconnectedness of medical, nursing and further treatment and support is accepted on paper, but not yet realized in actual care in Germany [34].

One study, analyzing hospitalizations for AC-SCs in a general population at high risk of re-hospitalization, also deemed the non-availability of the treating GP after office hours to be under the most important factors for potentially avoidable hospitalizations. Overprotective caregivers and the failure to use provided ambulatory services were other reasons [18]. These findings support our findings of the non-availability of the GP, overprotecting (in-)formal caregivers (fearing legal consequences) and hospitalizations for examinations/treatments also available in ambulatory settings contributing to the high number of hospitalizations of people with dementia.

Some interviewees, mainly GPs, stated that, in their opinion, the understaffing of some nursing homes and the respective lack of qualification of the nursing staff, contribute to hospitalizations. Porrell and Carter [35] show that staffing levels influence discretionary hospitalizations of people with dementia living in nursing homes. There is an objective shortage of qualified (wo-)manpower in nursing homes, it is one problem area identified by the Advisory Council on the Assessment of Developments in the Health Care System in Germany [36]. Our findings underscore that increased staffing levels in general, but also for increased levels of certified nursing staff in particular, could help to reduce hospitalizations.

### Implications for future research

As our study was retrospective and relied on the statements of members of the ambulatory health care system, further research is needed to explore the notion of preventability of hospitalizations of persons with dementia. Asking admitting physicians in hospitals, about their perception of the preventability or necessity of the hospitalization of patients with dementia at admission, might shed light on possible strategies to prevent certain kinds of hospitalizations. It could also quantify the amount of potentially preventable hospitalizations (especially for AC-SC) better than studies relying on claims data. A comparison between different countries would also reveal interesting details about the differences in healthcare systems regarding potentially preventable hospitalizations.

### Implications for policy makers

Ideas for reduction of hospitalizations were better qualified nurses, a 24-h-GP-emergency service and better compensation of ambulatory monitoring/treatments and house calls. Increasing the (dementia-specific) qualification and number of nurses might help to reduce hospitalization rates amongst people with dementia. Increased knowledge about medical conditions and characteristics of persons with dementia (for example as described above) can increase the motivation to use a “wait and see”- or try-ambulatory-treatment-first-strategy concerning conditions sensitive to ambulatory care. Blödt et al. [34] also recommend broader target-group-oriented offers for qualification relating to dementia.

There are insular initiatives like the “Alsterpflegenetz” (APN) in Hamburg (Germany) set up by the association of statuary health insurance physicians Hamburg (Kassenärztliche Vereinigung Hamburg, KVH). This is an example of a way to provide 24 h–GP-emergency service at least for people living in a nursing home. Besides this emergency service, communication between nurses and GPs is furthered in the APN by providing weekly consultation hours and visitations accompanied by a nurse from the nursing home [37]. This initiative aims directly at increasing the communication between nurses and GPs, securing a balanced and economic drug therapy and reducing hospitalizations. Initiatives like this should be expanded.

The subjective lack of reimbursement for extensive ambulatory care (including house calls to monitor treatments or the course of a disease) sometimes leads GPs to admit patients to hospitals instead of treating them in their homes or other ambulatory settings. Better reimbursement for monitoring house calls or short term support by a nursing service might have the potential to reduce hospitalizations. Another possibility might be the enhancement of telemedicine for patient monitoring [18].

## Conclusions

The prevention of the hospitalization of people with dementia, especially for ambulatory care sensitive conditions, has been constantly discussed for years. Evidence, from this explorative qualitative study and other epidemiologic studies, suggests that not all causal factors can be controlled by (primary) care providers (e.g. [38]). Reasons for potentially avoidable hospitalizations of people with dementia seem to be somewhat similar to those for unimpaired people. To gain clarity concerning the avoidabilty of potentially avoidable hospitalizations, further studies are needed, for example, observational studies in nursing homes to explore the processes of decision making about hospitalizations by (licensed and unlicensed) nurses in actual, live situations. Other studies could explore the admitting, hospital physicians’ evaluation of the avoidability of hospitalizations.

There is evidence that hospital staff feel that it has received insufficient training in caring for people with dementia [39]. Hospitals are often not suited for the special needs of people with dementia [40, 41]. Even if some of the hospital admissions of people with dementia are preventable, there is a strong need for hospital wards suited for people with dementia in order to reduce the negative impact of hospitalizations [40, 42]. In Germany (2010), only 12 geriatric departments/clinics had specialized wards for patients with cognitive impairments [43]. Dementia-specific training for hospital staff needs to be established and expanded [44]. Given that geriatric rehabilitation is effective in people with dementia (e.g. [45]), these programs should be offered to patients with dementia more often following a hospitalization.

## Additional files

Additional file 1: Interview guidelines. (PDF 43 kb)

Additional file 2: Table S1. Examples for hospitalizations and interviewees‘ views on preventability. Table showing summarized examples of hospitalizations and interviewees’ views on preventability. (DOCX 17 kb)

## Abbreviations

AC-SC: Ambulatory care-sensitive condition
APN: Alsterpflegenetz
GP: General practitioner
KVH: Kassenärztliche Vereinigung Hamburg (association of statuary health insurance physicians in Hamburg)
