# Supplementary material for: (In-)formal caregivers’ and general practitioners’ views on hospitalizations of people with dementia - an exploratory qualitative interview study
Source: BMC Health Serv Res. 2017 Aug 4;17:530. doi: 10.1186/s12913-017-2484-9 (PMC5545047; doi:10.1186/s12913-017-2484-9)
Supplement: Supplementary file 1 — Interview guidelines. (PDF 43 kb) [file 12913_2017_2484_MOESM1_ESM.pdf]

Guideline version 1: Informal caregiver (home care)

You care for Mr./Mrs. XY. Tell me about a day in the life of Mr./Mrs. XY.
Mr./Mrs. XY was in the hospital some time ago. How did it happen that Mr./Mrs. XY was admitted to the hospital?
Tell me about the state of Mr./Mrs. XY's physical and mental health.<br>In what state was Mr./Mrs. XY before his/her last hospital stay?
What do you usually do when you notice that Mr./Mrs. XY is not feeling well?<br>Whom do you speak with?
What are you missing in caring for Mr./Mrs. XY?
Was Mr./Mrs. XY's hospital stay necessary? In your opinion, how could the hospital admission have been avoided?
Do you want to add something else? Is there something important we have not talked about?

Guideline version 2: Informal caregiver (nursing home)

Mr./Mrs. XY lives in a nursing home. Tell us what you have to do for and with Mr./Mrs. XY in a typical week.
Mr./Mrs. XY was in the hospital some time ago. How did it happen that Mr./Mrs. XY was admitted to the hospital?
Tell me about the state of Mr./Mrs. XY's physical and mental health.<br>In what state was Mr./Mrs. XY before his/her last hospital stay?
What do you usually do when you notice that Mr./Mrs. XY is not feeling well?<br>With whom do you speak?<br>What happens if Mr./Mrs. XY is not feeling well?
Do you speak with the professional caregivers about Mr./Mrs. XY's state of health? How does that usually work? Would you give an example?
How is the contact between the professional caregivers/nursing home and the general practitioner?
What are you missing in the care of Mr./Mrs. XY?
Was Mr./Mrs. XY's hospital stay necessary? In your opinion, how could the hospital admission have been avoided?
Do you want to add something else? Is there something important we have not talked about?

Guideline version 3: General practitioners (home care)

Tell me about the state of Mr./Mrs. XY's physical and mental health.<br>In what state was Mr./Mrs. XY before his/her last hospital stay?
Describe your relationship to Mr./Mrs. XY.
Mr./Mrs. XY is cared for at home. Which topics do you talk about with Mr./Mrs. XY's caregiving relatives?
Has an emergency plan been made with the relatives in case Mr./Mrs. XY becomes ill? What does this plan look like?
Mr./Mrs. XY was in the hospital some time ago. How did it happen that Mr. /Mrs. XY was admitted to the hospital?
How would you assess the hospital admission(s) regarding its/their necessity? Would you admit Mr./Mrs. XY to the hospital if the same situation came up again? If not, why not?<br>If considered unnecessary: How could the hospital admission have been avoided in your opinion? Is there anything one could have done differently?
In your opinion, what is missing in the care for Mr./Mrs. XY?
Do you want to add something else? Is there something important we have not talked about?

Guideline version 4: General practitioner (nursing home)

Tell me about the state of Mr./Mrs. XY's physical and mental health.<br>In what state was Mr. /Mrs. XY before his/her last hospital stay?
Describe your relationship to Mr./Mrs. XY.
Mr./Mrs. XY is cared for in a nursing home. What kind of contact takes place between you and the nursing home /nursing staff?
How would you describe the general handling of hospital admissions through the nursing home?
Which topics do you talk about with the nursing home/nursing staff?
Which topics do you talk about with Mr./Mrs. XY's relatives about?
Has an emergency plan been made with the relatives or the nursing home in case Mr./Mrs. XY becomes ill? What does this plan look like?
Mr./Mrs. XY was in the hospital some time ago. How did it happen that Mr. /Mrs. XY was admitted to the hospital?
How would you assess the hospital admission(s) regarding its/their necessity?<br>Would you admit Mr./Mrs. XY to the hospital if the same situation came up again? If not, why not?<br>If considered unnecessary: How could the hospital stay have been avoided in your opinion?<br>Is there anything one could have done differently?
In your opinion, what is missing in the care of Mr./Mrs. XY?
Do you want to add something else? Is there something important we have not talked about?

Guideline version 5: formal caregiver (nursing home)

You work here in the nursing home. Tell us about a typical work day caring for Mr./Mrs. XY.
Who comes to visit Mr./Mrs. XY in the nursing home?<br>With which of Mr./Mrs. XY's relatives are you in contact?<br>What kind of contact takes place between yourself and Mr./Mrs. XY's relatives?
Which topics do you speak about when speaking with Mr./Mrs. XY's relatives?
Mr./Mrs. XY's also has an attending general practitioner. What kind of contact takes place between you and the general practitioner?<br>When and how often does the general practitioner come? What happens if a resident needs a physician between planned visits?
Which topics do you talk about with the general practitioner?
Does the general practitioner do more house calls on Mr. /Mrs. XY or does he/she generally go to the practice?
Tell me about the state of Mr./Mrs. XY's physical and mental health.<br>In what state was Mr./Mrs. XY before his/her last hospital stay?
Mr./Mrs. XY was in the hospital some time ago. How did it happen that Mr. /Mrs. XY was admitted to the hospital?
Was Mr./Mrs. XY's hospital stay necessary?<br>In the same situation, would you allow Mr./Mrs. XY to be admitted to the hospital again? If not, why not?<br>If considered unnecessary: How could the hospital stay have been avoided in your opinion?
How do you usually deal with Mr./Mrs. XY's illnesses or those of other nursing home residents?<br>When do you decide to call a doctor?
In your opinion, what is missing in the care of Mr./Mrs. XY?
Do you want to add something else? Is there something important we have not talked about?
