# Supplementary material for: (In-)formal caregivers’ and general practitioners’ views on hospitalizations of people with dementia - an exploratory qualitative interview study
Source: BMC Health Serv Res. 2017 Aug 4;17:530. doi: 10.1186/s12913-017-2484-9 (PMC5545047; doi:10.1186/s12913-017-2484-9)
Supplement: Supplementary file 2 — Examples for hospitalizations and interviewees‘ views on preventability. Table showing summarized examples of hospitalizations and interviewees’ views on preventability. (DOCX 17 kb) [file 12913_2017_2484_MOESM2_ESM.docx]

Supplementary Table 1: Examples for hospitalizations and interviewees‘ views on their preventability

| **Informal caregiver** | | **General practitioner (GP)** | | **Formal caregiver** | |
| --- | --- | --- | --- | --- | --- |
| **description of hospitalization** | **View on preventability/reasons for non-preventability** | **description of hospitalization** | **View on preventability/reasons for non-preventability** | **description of hospitalization** | **View on preventability/reasons for non-preventability** |
| **Planned treatments/operations** | | | | | |
| allergic skin reaction, where ambulant treatment by both the GP and dermatologist did not help | not preventable, rather late admission to the hospital | eczema, treatment by GP and dermatologist not sufficient, admission by GP | not preventable, eczema has been treated unsuccessfully by GP and dermatologist (ambulant care) | nurses discovered skin changes that were treated unsuccessfully by the GP, thus, treatment in hospital | not preventable, due to unsuccessful ambulatory treatment |
| **Unplanned (emergency) treatments due to aggravation of the general condition or exsiccosis** | | | | | |
| a nursing home resident found the person with dementia on the floor and alarmed a nurse, the person with dementia was transferred to the hospital, pulse was very slow, blood pressure very low | necessary hospitalization, if something like that happens a physician is promptly needed to make sure that everything is alright | ^a^ the person with dementia collapsed due to exsiccosis, pulse was very slow, blood pressure very low | not preventable, intravenous replacement of fluids only possible in the hospital and better than subcutaneous | no interview | no interview |
| **falls (nursing home)** | | | | | |
| Unobserved fall, nurses called the ambulance upon finding the person with dementia | - | ^a^ fall, fracture | not preventable, risk of falls is always given, person with dementia cannot be watched around the clock | no interview | no interview |
| **falls (at home)** | | | | | |
| The person with dementia left the outpatient care service on its own and fell, was transferred to the hospital for ambulatory treatment of the wounds, further treatment took place in a stationary setting because of the person’s blood thinning medications | - | the GP had no information about this incidence | - | The person with dementia left the outpatient care service on its own and was searched for by different persons, the person with dementia fell down, passers-by called an ambulance, because the person with dementia was injured | not preventable, free movement of outpatients may not be restricted, tendencies to stray were not strongly apparent in the person with dementia |
| **other reasons** | | | | | |
| the person with dementia observed blood in the stool and activated the emergency button, the nurses called an ambulance | not preventable | ^a^ diverticulitis, can cause massive bleeding | not preventable, complications can occur, person needs to be examined and treated in hospital | no interview | no interview |
| the person with dementia’s child called an ambulance, because the person with dementia was vomiting repeatedly and complained about pain (after GP’s office hours) | not preventable | ^a^ abdominal influenza, exsiccosis | hospitalization was unnecessary, could have been treated with a high fluid intake at home | no interview | no interview |

^a^ = Information from discharge letter (not referred by GP)¸- = no information provided during the interview
